# Acoustically Triggered Disassembly of Multilayered Polyelectrolyte Thin Films through Gigahertz Resonators for Controlled Drug Release Applications

**DOI:** 10.3390/mi7110194

**Published:** 2016-11-01

**Authors:** Zhixin Zhang, Zifan Tang, Wenpeng Liu, Hongxiang Zhang, Yao Lu, Yanyan Wang, Wei Pang, Hao Zhang, Xuexin Duan

**Affiliations:** State Key Laboratory of Precision Measuring Technology & Instruments, Tianjin University, Tianjin 300072, China; zxzhang10@tju.edu.cn (Z.Z.); zifantang@tju.edu.cn (Z.T.); liuwenpeng@tju.edu.cn (W.L.); tjzhx@tju.edu.cn (H.Z.); luyao@tju.edu.cn (Y.L.); yanyanwang@tju.edu.cn (Y.W.); weipang@tju.edu.cn (W.P.); haozhang@tju.edu.cn (H.Z.)

**Keywords:** controlled drug release, layer-by-layer (LbL), bulk acoustic resonator, MEMS

## Abstract

Controlled drug release has a high priority for the development of modern medicine and biochemistry. To develop a versatile method for controlled release, a miniaturized acoustic gigahertz (GHz) resonator is designed and fabricated which can transfer electric supply to mechanical vibrations. By contacting with liquid, the GHz resonator directly excites streaming flows and induces physical shear stress to tear the multilayered polyelectrolyte (PET) thin films. Due to the ultra-high working frequency, the shear stress is greatly intensified, which results in a controlled disassembling of the PET thin films. This technique is demonstrated as an effective method to trigger and control the drug release. Both theory analysis and controlled release experiments prove the thin film destruction and the drug release.

## 1. Introduction

Every drug molecule has a therapeutic window in terms of the concentration above which the drug is toxic and below which it is ineffective [1]. Developing controlled drug release techniques to precisely tune the dosage of drugs is a critical issue in modern medical and biotechnology research [2,3,4]. Many research interests are focused on developing a system based on molecular self-assembly approaches as novel drug carriers to realize controlled drug release [5,6], where the drug molecule is slowly released over time. Among these dedicated chemical approaches, drug carriers based on the self-assembly of polyelectrolytes (PETs) through layer-by-layer (LbL) techniques are one of the most promising methods, in which multilayered thin film is established by alternating the assembly of oppositely charged PETs on surfaces or in solutions [7,8,9]. Drug molecules can be incorporated directly during the LbL assembly process or absorbed into the nanoporous PET films after the LBL approach [10]. It is a very simple and reliable technique as the architectures of the thin films can be well controlled at nanometer precision; thus, the amount of the drug molecules loaded can be precisely determined. The drug release can be controlled by an external stimulus to trigger the disassembly of the PETs, such as pH [11,12,13], electrochemistry [14,15,16], temperature [17,18,19], or light [20,21,22]. However, these methods are limited by either chemical properties of the PET systems or specifications on the operating environment. For practical applications, it is desired to develop methods which are not limited by the chemical properties of the system or the environment, and with low power consumption.

Owing to their small size and high precision, micro-electromechanical systems (MEMS) technologies are thought to be a potential candidate to realize controlled release. Micro-devices such as the microneedle [23,24,25] and micropump [26,27,28] have been developed for this purpose. In this work, we developed a novel versatile controlled drug release method which utilizes a miniaturized MEMS acoustic resonator to excite streaming flows and physical shear stress which acts as a tearing force to trigger the disassembly of the multilayered PET thin films. The resonator transfers an electric supply to the mechanical vibrations of the gigahertz (GHz), which greatly strengthens the shear stress due to the ultra-high frequency of the vibration. The input power required is around several hundreds of milliwatts (mW), which is a low power consumption. Additionally, owing to the miniature size of the device, the resonator opens the possibilities of being implanted in vivo to realize site-targeted delivery.

## 2. Materials and Methods

### 2.1. Materials

Sodium hyaluronate (HA), Poly-L-lysine (PLL), Poly-L-lysine-FITC (PLL-FITC), *N*-hydroxysulfosuccinimide sodium salt (sulfoNHS), and 1-ethyl-3-(3-dimethylaminopropyl) carbodiimide hydrochloride (EDC) are purchased from Sigma-Aldrich (San Antonio, TX, USA). Doxorubicin hydrochloride (DOX·HCl) is obtained from Aladdin (Shanghai, China).

### 2.2. Device Fabrication

The device is fabricated through standard MEMS process (shown in the Supplementary Figure S1). The process starts from deposition of brag reflection layers on silicon substrate, and the reflectors are composed of 1.2 μm AlN, 0.7 μm SiO_2_, 1.0 μm AlN, 1.3 μm SiO_2_, 1.0 μm AlN, 0.65 μm SiO_2_, from bottom to up. Thin film of molybdenum (Mo) was deposited on stacked reflector substrates using RF sputtering. After that, Mo was patterned as the bottom electrode by plasma etching. Then, AlN and Mo were deposited in turn onto the bottom electrode. Wet etching was then used to pattern the top electrode using the mixed etchant of nitric acid, acetic acid, and phosphoric acid. Subsequently, AlN was etched by a combination of plasma etching and potassium hydroxide (KOH) wet etching. After AlN etching, the bottom electrode can be accessed. Finally, Gold (Au) was evaporated and patterned by lift-off process, serving as electrical connections and testing pads.

### 2.3. Polyelectrolyte Multilayers Preparation and DOX Loading

Polyelectrolyte multilayers were prepared by alternating adsorption of PLL and HA on the surface of resonators or glass substrates from 2 mg/mL PLL to 5 mg/mL HA aqueous solutions in the presence of 0.15 M NaCl (pH = 6.5) and at 15 min of incubation time for each layer. The total number of polymer layers was 12 for each sample. The polymer films were coated on the whole resonator, both the active area and the non-active area of the device. The thin films were immersed in the DOX solution (5 mg/mL, in the presence of 0.15 M NaCl) for 12 h at 37 °C. Chemical cross-linking of the multilayers was performed by incubating the thin films in the EDC/sulfo-NHS solution (pH 6.5) overnight. The concentration of the EDC and the sulfo-NHS was 400 mM and 50 mM, respectively.

### 2.4. Release System

A chamber is sealed on the top of the resonator, which was coated with multilayered polyelectrolyte thin films. The chamber was filled with buffer solution (0.15 M NaCl aqueous solution, typical 100 μL). Sinusoidal signal of 1.5 GHz (intrinsic resonant frequency of the resonator) was generated by a signal generator (N5181A, Agilent, Santa Clara, CA, USA), amplified by a power amplifier (ZHL-5W-422, Mini-Circuits, New York, NY, USA), and sent to the resonator, which then transduced the electrical supply to mechanical vibrations, and further induced fluidic flow in the liquid.

### 2.5. Assessment of the DOX Release

Fluorescence microscope (BX53, Olympus, Center Valley, PA, USA) was used to examine the fluorescence intensities of DOX left in the multilayers. To quantify the DOX release, the fluorescence intensities of the buffer which dissolved the released DOX was measured by a fluorescence spectrophotometer (Varioksan LUX, Thermal Scientific, Beaumont, TX, USA), at Ex 485 nm and Em 550 nm.

### 2.6. SEM Characterization of the Thin Films

To investigate the thin films disassembly, the scanning electron microscope (SEM, FEI F50, Houston, TX, USA) was utilized to characterize the morphology of the multilayers. The films on the surface of the device (or glass) was washed by the buffer, and then dried under nitrogen. Finally, the carrier sample was examined with the SEM.

## 3. Results

### 3.1. Theory Consideration

**Figure 1:**
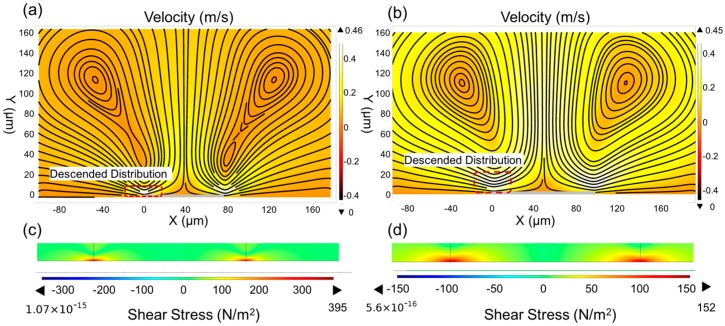
Simulated results of the 2D FEM analysis. Micro-vortices are formed under the resonator stimulation of (**a**) 1.5 GHz and (**b**) 750 MHz. The simulated shear stress induced by the flow with the excitation frequency of (**c**) 1.5 GHz and (**d**) 750 MHz.

It is known that when the acoustic resonator works in liquid, the vibration of the resonator will trigger the micro-streaming in the liquid [29], providing a local flow condition. To investigate the fluid behaviors triggered by the gigahertz resonator, we applied a two-dimensional finite element model (2D FEM) to analyze the patterns and the velocities of the flow. As the flow trigger, the resonator was modeled at the bottom of the liquid. The simulation results are shown in Figure 1. Two symmetric micro-vortices are clearly observed under the vibration stimulus. The liquid near the edge of the device ejects upward and returns from the periphery, forming the flow circulation. The simulated color patterns indicate the velocity distributions in the liquid. The device located in the streaming hinders the flow and decreases the velocity. Thus, in the micro-sized area above the device, the velocity expresses the descended distribution from top to bottom, and approaches zero at the surface of the device. The vertical gradient of the velocity produces shear stress, which acts as a tearing force at the solid-liquid interface. Following Newton’s viscosity law, the shear stress is proportional to the amplitude of the gradient.

To further understand the relationship between the shear stress and the resonant frequencies, we simulated the flow conditions under the resonant frequencies of 1.5 GHz (Figure 1a) and 750 MHz (Figure 1b). It clearly shows that by levitating the frequency of the excitation, though the velocities remain the same, the vortex contracts in the vertical direction, and intensifies the vertical gradient of the velocity, thus leading to an enhancement of the shear stress. The simulated shear stress induced by streaming flows is shown in Figure 1c,d. The results show that the shear stress increases with the exciting frequency, which confirms the above theoretical predictions. It also indicates that the high-resonant-frequency device will induce a much stronger fluid flow and further increase the shear stress, which can be used as the driving force to manipulate the thin films assembled at the liquid-solid interface.

### 3.2. Triggered PET Surface Disassembly

**Figure 2:**
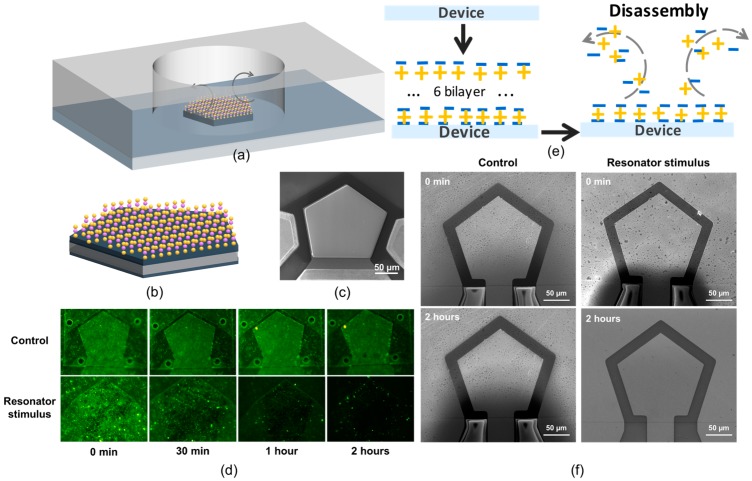
(**a**) Schematic of the release system. The streaming flow was triggered by the stimulation of the resonator, inducing shear stress on the solid-liquid interface, and further leading to the disassembly of PET thin films; (**b**) Multilayered PET-coated resonator composed of HA and PLL-FITC through LbL approach; (**c**) SEM image of the fabricated resonator, which is comprised by a thin-film piezoelectric layer sandwiched by two metal electrodes and vibrates at 1.5 GHz; (**d**) Fluorescence images of the PET thin films, which were exposed to the buffer with (experiment group) or without (control group) the stimulation of the resonator; (**e**) Schematic process of the multilayers’ disassembly under the stimulation of the resonator; (**f**) SEM images of the PET thin films before and after the treatment.

According to the FEM analysis, the stress induced by the high-frequency resonator can directly interact with the thin films at the liquid-solid interface and contribute to their disassemblies. To demonstrate the concept, we designed and fabricated a bulk acoustic resonator composed of a thin piezoelectric layer sandwiched by two metal electrodes which vibrates at 1.5 GHz. The device is fabricated through a Complementary Metal Oxide Semiconductor-compatible (CMOS-compatible) process. Figure 2c shows the scanning electron microscope (SEM) image of the fabricated device. To maximize the utilization efficiency of energy, a polygon-shaped resonator is designed which can focus more energy in the main working mode.

A reaction chamber–based drug release setup was built up for the convenience of drug release and detection (Figure 2a). A chamber was sealed on a multilayered polyelectrolyte-coated device which was filled with buffer solution. The amplified sinusoidal signal of 1.5 GHz was sent to the device, transducing the electrical supply to mechanical vibrations and exciting a swift flow in the liquid. The electric input is conveniently switched on and off using a program control, and thus the treatment time can be well controlled. Since the released drug molecules can dissolve in the buffer, the amount of the released drug can be quantified by the fluorescence technique either from the buffer or on the surface.

To demonstrate the controlled molecular surface disassembly, thin films of sodium hyaluronate (HA) and Poly-L-lysine (PLL) were used as a template polyelectrolyte (PET) assembly system. HA is a negatively charged PET while PLL is positively charged; thus, they could assemble on the substrate in a layer-by-layer (LbL) fashion and form stable PET thin films. In order to facilitate the visualization and quantification of the PET disassembly, the PLL was labeled with FITC; thus, the disassembly process can be directly evaluated through fluorescence detections. Six bilayers of HA and PLL-FITC were first coated on the resonator surface. After thoroughly cleaning with the buffer, the device was fixed in the reaction chamber filled with pure buffer solutions and 640 mW power was applied to the resonator to trigger the PET disassembly, while another device coated with the same HA and PLL-FITC was immersed in the same buffer system without stimulation of the resonator as a control group. The fluorescence intensities of the thin films were checked at 0, 30, 60, and 120 min, and the corresponding images are shown in Figure 2d. No appreciable deduction of the florescence intensity was detected in the control group which indicates the stability of the PET thin films in the buffer system, while in the experiment group, the fluorescence intensity clearly decreases with time. The result demonstrates that the disassembly of the PET thin films only occurs in the presence of the resonator stimulations, which further indicates that the resonator can effectively trigger the destruction of the PET thin films, as schematically shown in Figure 2e. In addition, there was little fluorescence left on the device after the treatment of 2 h, suggesting the resonator stimulus has successfully disassembled most of the PETs.

To further ensure the destruction of the PETs, the scanning electron microscope (SEM) was utilized to characterize the morphologies of the thin films. As shown in Figure 2f, no obvious changes take place in the control group. In contrast, most of the PETs have been removed from the surface after the device simulation, which indicates the success of the disassembly. All the results agree well with the above fluorescent characterizations.

### 3.3. Controlled DOX Release

As demonstrated above, the GHz resonator can be used as an effective source to trigger the PETs’ surface disassembly. Since the PET assembly system can be used to entrap the drug molecules [3,30], the demonstrated acoustic triggered disassembly can be utilized as an effective technique to activate the drug release. As shown in Figure 2d, the fluorescence intensity decreased gradually under the acoustical stimulus, suggesting that the PET thin films disassemble little by little. This can be used for sustained drug release applications, which is one of the most important issues in drug delivery. Further experiments were carried out to study the kinetics of the drug release under the stimulations of different input powers.

**Figure 3:**
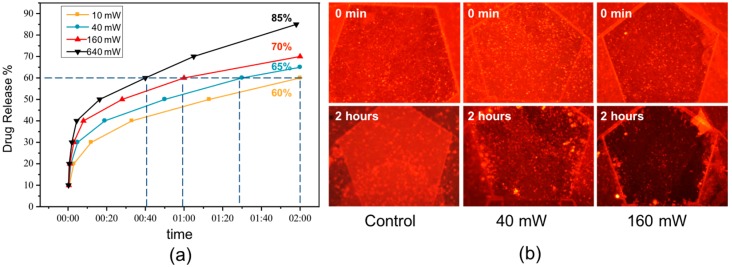
(**a**) DOX release under the stimulus of the resonator with different power inputs; (**b**) Fluorescence images of the thin films taken before and after the experiments.

Doxorubicin (DOX) was chosen as the model drug and was trapped in PET thin films by immersion of the films in the DOX solution (5 mg/mL, in the presence of 0.15 M NaCl) for 12 h at 37 °C. Since the released DOX is dissolved in the buffer, the amount of the released drug can be quantitatively monitored by detecting the fluorescence intensities of the buffer (Figure 3a). According to the results, it clearly showed that the amount of the released DOX increases with treatment time for all power supplies, which coincides with the results from the HA-PLL surface disassembly. However, different input power leads to different percentages of DOX release. Higher power corresponds to a larger amount of release, showing that the release progress is input power–dependent. It further indicates that the amount of the release can be well controlled by tuning the input power. Similarly, to reach the same target amount of release, the required stimulation time can also be controlled through the adjustment of the input power. Taking 60% DOX release as an example, the required stimulation time with an input power of 10 mW is 120 min. Increasing the power amplitude to 40, 160, and 640 mW, the required treatment time decreases to 90, 60, and 40 min, respectively.

To further prove the DOX release, fluorescence images of the PET thin films were taken before and after the experiments (Figure 3b). Compared with the control group, the experiment groups exhibit an obvious fluorescence deduction after the acoustic stimulation, which demonstrates the successful release of DOX from the carrier. It also shows a clear difference between the two experiment groups with the input power of 40 mW and 640 mW. The latter group emits much less fluorescence than the former, confirming that much more DOX has been released under the stimulation of the higher input power.

All these results indicate that the drug release triggered by the resonator is a power-dependent process, and the stronger power will lead to a higher release rate. This also can be explained in view of the flow velocity. Increasing the input power accelerates the velocity of the flow, which enhances the vertical gradient of the velocity, thus strengthening the shear stress, and further speeds up the drug release.

## 4. Discussion

**Figure 4:**
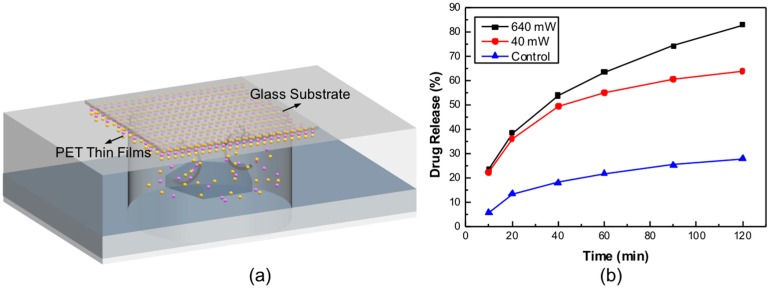
(**a**) Schematic of the release system where the PET thin films were coated on a piece of glass and fixed above the device 2 mm away; (**b**) DOX release with time under the stimulus of the resonator. The input power is 640 mW.

As shown above, the main driving force to trigger the PET surface disassembly/DOX release is the tear force from the high-speed micro-vortex at the solid-liquid interface induced by the GHz resonator. In principle, such force can not only work on the films assembled on the device surface, but also on other substrates which are able to contact the micro-vortex. To demonstrate this, the same multilayered PET thin films (HA-PLL) incorporated with DOX were coated on a piece of glass and fixed above the resonator at a distance of 2 mm, by leaving the PET films toward the resonator. Then, 640 mW power was continuously applied to the device for 2 h. The input power of 640 mW can heat the liquid from room temperature to around 37 °C. To exclude the impact of the temperature on the film disassembly, the control experiment was also conducted using a 37 °C solvent. The fluorescent intensities of the released DOX were measured every 20 min and plotted in Figure 4b. It clearly shows that up to 90% of DOX has been released, showing the successful controlled release which is triggered by the acoustic resonator. In contrast, only a little DOX is released in the control group, suggesting the heat effect on liquid has little impact on film stability. The results indicate that the acoustically triggered disassembly of multilayered polyelectrolyte thin films through gigahertz resonators is a versatile method for controlled drug release applications not only for carriers on the device surface, but also on other substrates close to the device, thus increasing the throughput of this technique. In addition, owing to the miniature size of the resonator, it opens possibilities to be implanted in vivo to realize site-targeted delivery.

## 5. Conclusions

In summary, we have developed a novel versatile physical technique to trigger disassembly of multilayered polyelectrolyte thin films for controlled drug release applications which utilizes the GHz bulk acoustic resonator to excite the local micro-vortex and further induce shear stress at the solid-liquid interface. The ultra-high frequency of the resonator enhances the vertical gradient of the flow velocities, and therefore intensifies the shear stress, making it a powerful source to tear and disassemble the PET thin films. Due to the moderate physical interactions between the flow and the films, the multilayers disassemble step by step; thus, sustained drug release can be achieved in this manner. The input power required is around several hundreds of milliwatts (mW), indicating the method requires low power consumption. Owing to the intrinsic miniature size of the resonator, it opens possibilities for implantation in vivo to realize site-targeted delivery. In addition, the shear wave mode can be considered as a future development of the proposed application due to the direct generation of shear stress during wave propagation.

## Supplementary Materials

The following are available online at www.mdpi.com/2072-666X/7/11/194/s1, Figure S1: Schematic of the fabrication process of hypersonic resonator. (**a**) A silicon substrate is prepared; (**b**) Brag reflector layers are deposited in turn on the substrate; (**c**) Molybdenum (Mo) is deposited and patterned as the bottom electrode; (**d**) Aluminum nitride (AlN) is deposited as a piezoelectric layer; (**e**) Mo is deposited and patterned as the top electrode; (**f**) AlN is etched, and gold (Au) is deposited and patterned as the connecting and testing pad.
