# Supplementary material for: Acoustically Triggered Disassembly of Multilayered Polyelectrolyte Thin Films through Gigahertz Resonators for Controlled Drug Release Applications
Source: Micromachines (Basel). 2016 Nov 1;7(11):194. doi: 10.3390/mi7110194 (PMC6189713; doi:10.3390/mi7110194)
Supplement: Supplementary file 1 [file micromachines-07-00194-s001.pdf]

Supplementary Materials: Acoustically Triggered Disassembly of Multilayered Polyelectrolyte Thin Films through Gigahertz Resonators for Controlled Drug Release Applications

Zhixin Zhang, Zifan Tang, Wenpeng Liu, Hongxiang Zhang, Yao Lu, Yanyan Wang, Wei Pang, Hao Zhang and Xuexin Duan

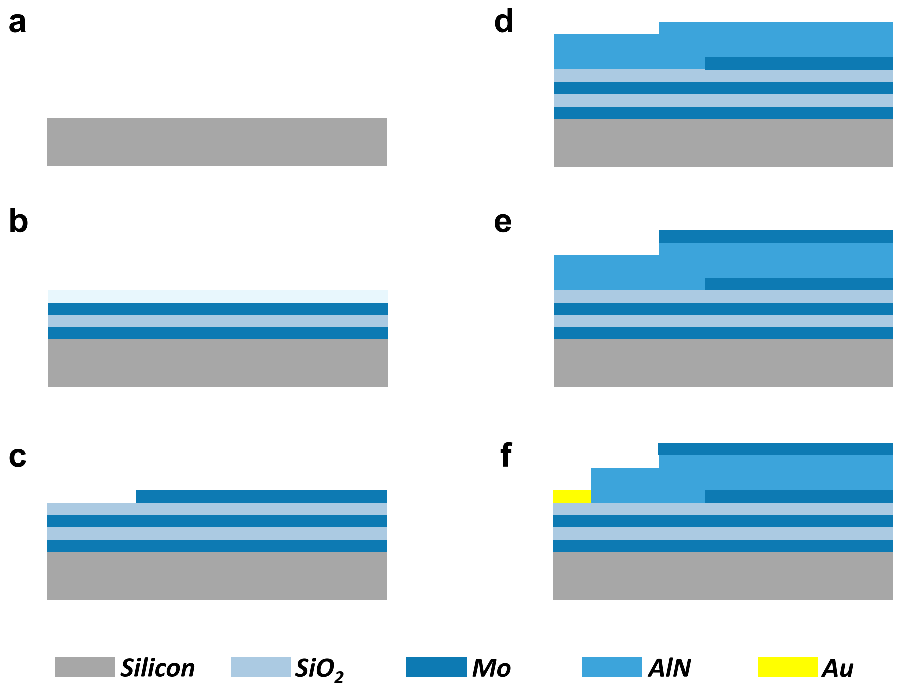

Figure S1. Schematic of the fabrication process of hypersonic resonator. (**a**) A silicon substrate is prepared; (**b**) Brag reflector layers are deposited in turn on the substrate; (**c**) Molybdenum (Mo) is deposited and patterned as the bottom electrode; (**d**) Aluminum nitride (AlN) is deposited as a piezoelectric layer; (**e**) Mo is deposited and patterned as the top electrode; (**f**) AlN is etched, and gold (Au) is deposited and patterned as the connecting and testing pad.
